# Antimicrobial activity of low-pressure plasma treatment against selected foodborne bacteria and meat microbiota

**DOI:** 10.1007/s13213-014-0992-y

**Published:** 2014-11-19

**Authors:** Natalia Ulbin-Figlewicz, Andrzej Jarmoluk, Krzysztof Marycz

**Affiliations:** 1 Department of Animal Products Technology and Quality Management, Wrocław University of Environmental and Life Sciences, ul. Chełmońskiego 37/41, 51-630 Wrocław, Poland; 2 Department of Animal Hygiene and Animal Welfare, Wrocław University of Environmental and Life Sciences, ul. Chełmońskiego 38 C, 51-631 Wrocław, Poland

**Keywords:** Antibacterial activity, Bacterial cell, Cold plasma, Decontamination, Low-pressure plasma, Meat

## Abstract

The effects of helium and argon plasma treatments on inactivation of both pure bacterial cultures inoculated onto the surface of agarized media and the surface microbiota of meat were investigated. Cold plasmas were generated by high voltage discharge at low pressure (20 kPa) for 2, 5, and 10 min. The number of viable microorganisms was determined using a plate count method. Morphological changes were observed using scanning electron microscopy (SEM). Microbial log reduction depended on time of exposure and type of gas used. After a 10-min treatment with helium plasma, the total number of microorganisms, yeasts and molds, and psychrotrophic microorganisms was reduced in the range of 1.14–1.48 log cycles for pork and 0.98–2.09 log cycles for beef. A significant reduction of 2.00 log for *Bacillus subtilis* and *Yersinia enterocolitica* was achieved within 2 min of helium plasma treatment. Similar results were obtained for *Staphylococcus aureus*, *Escherichia coli a*nd *Pseudomonas fluorescens* after 5 min and 10 min of exposure. SEM revealed disruption and lysis of *E. coli* cells treated with helium plasma for 10 min, suggesting a bactericidal effect.

## Introduction

Microbial hazards are one of the most important issues in the food industry. Meat spoilage is caused by three basic mechanisms: microbial growth, lipid oxidation and enzymatic autolysis (Dave and Ghaly 2011). Those result in discoloration, slime formation, undesirable odors and flavors, and texture softening and make the product unacceptable to consumers. After slaughter and chilling, microbial counts on carcasses are in the range of 10^1^–10^5^ colony forming units (CFU)/cm^2^. The types and numbers of microorganisms depend on the initial microbiota, processing, and storage conditions. The most common sources of carcass meat contamination are *Pseudomonas* spp., *Moraxella* spp., *Acinetobacter* spp., *Alcaligenes* spp., *Flavobacterium* spp., *Aeromonas* spp., *Staphylococcus* spp., *Micrococcus* spp., coryneforms and *Enterobacteriaceae*. Meat may be also contaminated by bacterial pathogens, including *Salmonella* spp., *Campylobacter jejuni*/*coli*, *Escherichia coli*, *Listeria monocytogenes*, *Staphylocccus aureus*, *Yersinia enterocolitica*/*pseudotuberculosis*, *Bacillus cereus*, *Clostridium perfringens* and *Clostridium botulinum* (Sofos 1994; Borch et al. 1996). The most common spoilage microorganism of fresh chilled meat is *Pseudomonas* spp. These bacteria produce putrefactive odors and slime when their population exceeds 10^7^ CFU/cm^2^ (Sofos 1994).

The meat industry is an important part of the food manufacturing and processing sector. Between 1960 and 2000, there was a large increase in meat consumption worldwide. The annual per capita consumption increased from 10 kg to 26 kg (Dave and Ghaly 2011). In Europe, as a total, 23.8 % of meat and meat products are lost and wasted in the food supply chain. The main sources of loss and waste come from consumption (46 %), processing and packaging (21 %), distribution (17 %), production (13 %), handling and storage (3 %) (Kanerva 2013). Furthermore conventional preservation methods such as refrigeration and modified atmosphere packaging (MAP) technology could be insufficient in the case of fresh, unprocessed meat. Thus, researchers are looking for non-thermal technologies that will inactivate or inhibit growth of microbiota while maintaining meat quality (Zhou et al. 2010).

Many studies indicate that cold plasma treatment may be employed for the preservation of food (Moreau et al. 2008; Song et al. 2009; Kim et al. 2011). Cold plasma as an ionized gas contains UV photons, neutral atoms and molecules, excited atoms and molecules, electrons and free radicals (Moreau et al. 2008). Those plasma species are involved in the inactivation process by interacting with bacterial cells, causing: damage to DNA by UV irradiation (living cells cannot repair lesions of the DNA strands sufficiently quickly); erosion of the microorganism through intrinsic photodesorption (formation of volatile compounds due to breakages of chemical bonds in microbial material) and through etching involving free radicals (Moisan et al. 2002). Low-pressure plasma techniques have various advantages for use in the meat industry. They are environmentally friendly and work at low temperatures, so thermolabile products can be treated. The other benefit relates to the fact that plasma treatments can be applied to large areas (limited by vacuum chamber size), and can be distributed evenly inside the chamber so the whole surface of the material is treated uniformly. On the other hand, vacuum systems are expensive, thus economic aspects need to be considered (Schütze et al. 1998).

The aim of this study was to investigate antimicrobial activity of low-pressure argon and helium plasma treatment against bacterial species generally encountered in meat spoilage as well as against surface microbiota of pork and beef muscles.

## Materials and methods

### Antimicrobial activity

#### Pure bacterial cultures

Five strains of Gram-positive and three Gram-negative bacteria were selected as test organisms: *Microccocus luteus* PCM 1944, *Lactobacillus acidophilus* PCM 2510, *Bacillus subtilis* PCM 2021 (vegetative forms), *Staphylococcus aureus* PCM 2602, *Listeria monocytogenes* PCM 2606, *Escherichia coli* PCM 2560, *Yersinia enterocolitica* PCM 2080, and *Pseudomonas fluorescens* PCM 1994. These microorganisms were obtained from the culture collections of Institute of Immunology and Experimental Therapy, Polish Academy of Sciences. The bacteria selected are frequently reported in the literature as responsible for meat and meat product spoilage (Dave and Ghaly 2011).

Antibacterial activity was assessed using the plate method. *P. fluorescens*, *B. subtilis*, *S. aureus*, *E. coli*, and *Y. enterocolitica* inoculum were prepared by growing cells in enriched broth containing beef broth, peptone, sodium chloride, peptone C, yeast extract (BTL, Lodz, Poland) for 24 h at 37 °C and at 25 °C for *M. luteus. Lactobacillus acidophilus* were grown in MRS broth (Merck, Warsaw, Poland) at 37 °C and *Listeria monocytogenes* in BHI broth (Merck) at 30 °C. Optical density of bacterial cultures was measured using a spectrophotometer UV 1800 (Rayleigh Instruments, Rayleigh, UK) at 550 nm. Enumeration of bacteria in control samples was determined using the viable plate count method. Inoculum containing 10^4^ CFU/ml was diluted (1:10, 1:100) and 0.1 mL of final two dilutions was transferred to duplicate nutrient agar plates. The number of CFU/mL in the control sample can then be determined by multiplying the number of colonies on a dilution plate by the corresponding dilution factor. Only plates (or replicate plates from the same dilution) with 30–300 colonies were counted.

Plates that had been seeded previously with 0.1 mL inoculum containing 10^4^ CFU/mL test bacteria were exposed to helium and argon plasma treatment for 2, 5 and 10 min at low pressure (20 kPa) and then incubated as noted above. Initial populations in control samples were about 10^3^ CFU/mL. Results are expressed as log reduction and were calculated as shown in following equation:

1 $$ Logreduction= \log {}_{10}\left({N}_0\right)- \log {}_{10}(N) $$

where,

- N_0_ is the number of viable microorganisms before treatment (initial population)
- N the number of viable microorganisms after treatment.

#### Plasma treatments and microbial analysis of raw meat

Fresh pork longissimus dorsi muscle (24 h post mortem) and beef musculus semitendinosus muscle (48 h post mortem) were purchased from a local meat processing plant (Dworecki, Poland). The surface layers of muscle (thickness 2 cm, length 7 cm, width 7 cm) were cut out and exposed to helium and argon cold plasma for 2, 5 and 10 min at low pressure (20 kPa). The experiments were conducted in triplicate, using the same meat batch for each one. These samples were compared to samples not subjected to cold plasma treatment. Temperature of meat samples was measured immediately before and after low-pressure plasma treatment. The average temperature of untreated sample was 4.1 °C and increased to 7.0 °C after 10 min exposure. Sampling was started immediately after low-pressure plasma exposure. Sterile cotton swabs were used for surface sampling in accordance with ISO 18593:2004^1^. The swab head was moistened with sterile saline solution and excessive solution was pressed out against the interior wall of the vial with a rotating motion. A template with a 5 cm × 5 cm opening was used to sample the same surface area each time. After the area has been swabbed, the swab head was placed in the vial and series of dilutions were prepared (ISO 17604:2003)^2^. Determination of total number of microorganisms (TNM) was done using the plate method described in ISO 2293:1988^3^. Colonies were counted after 72 h of incubation at 30 °C on culture medium containing tryptone (BTL), yeast extract (Merck), glucose (BTL) and agar (Merck). Psychrotrophic microorganisms (P) were determined on PCA plates (hydrolyzed casein (BTL), yeast extract, glucose, and agar incubated at 6 °C for 10 days (ISO 17410:2001)^4^. Culture medium of yeast and mold (Y&M) was prepared using yeast extract, glucose, agar and was supplemented with antibiotic (chloramphenicol) (Sigma-Aldrich, Poland). Plates were incubated for 5 days at 25 °C (ISO 21527–1:2008)^5^. The CFU per milliliter of each sample was calculated as shown in the following equation:

2 $$ N={\displaystyle \sum C/}\left\{\left({n}_1\times 1\right)+\left({n}_2\times 0.1\right)\right\}d $$

where,

- N is the number of colonies per milliliter of the product (CFU/ml)
- ∑C is the sum of all colonies in all plates counted
- n_1_ is the number of plates in lower dilution counted
- n_2_ the number of plates higher dilution counted
- d is the dilution level corresponding to first count (n_1_)

The CFU per square centimeter were calculated using the following formula:

3 $$ {N}_s=\left(N\times F\right)/A $$

where,

- N is the number of colonies per milliliter of product (CFU/mL)
- F is the amount (mL) of dilution fluid
- A is the surface investigated (cm^2^).

The results are expressed as log reduction and were calculated according to Eq (1) above.

#### Low-pressure cold plasma treatment

**Fig 1:**
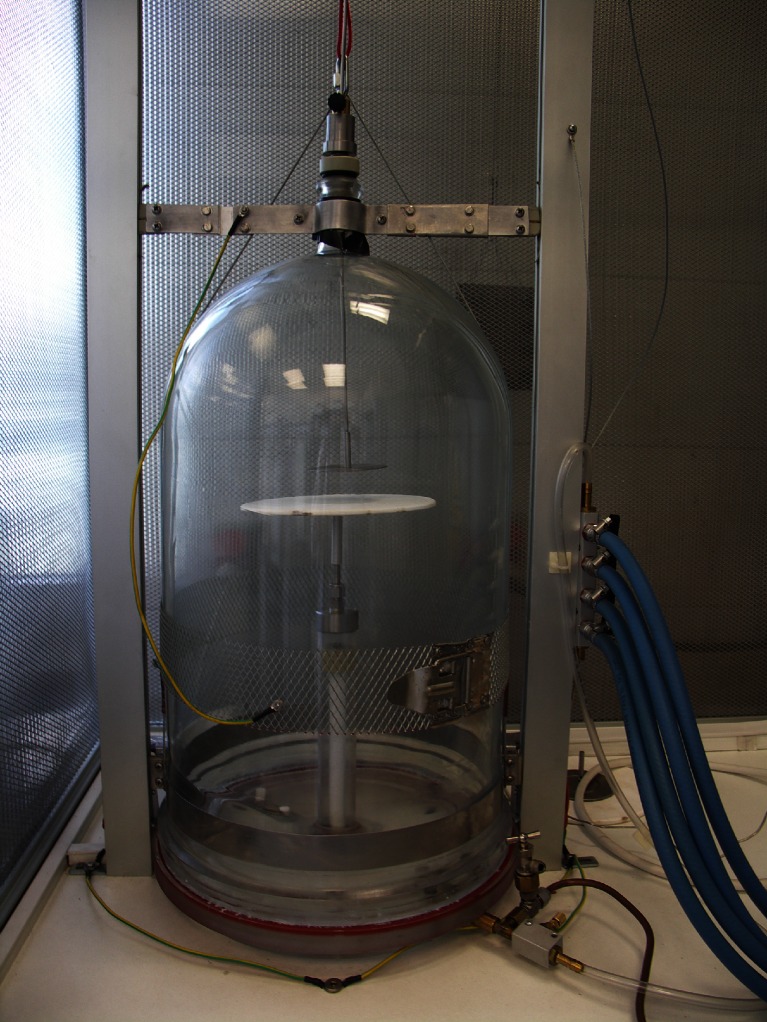
Prototype generator of cold plasma

The laboratory pulsed plasma reactor (Ertec Poland, Wrocław, Poland) used in this experiment is shown in Fig. 1. Cold plasma was generated by high voltage discharge in a vacuum chamber (diameter 250 mm, height 500 mm, vacuum 100 Pa) between the electrodes of the discharge condenser located on a high voltage table, where the sample was placed in glow position. The electrodes were made of quartz frits and the distance between them was 5 cm. Running pressure was 20 kPa. Gas flow was set at 3 L/min. The frequencies used for the alternating voltages were between 20 and 100 kHz and reactive power was 1.2 kVA. In contrast to atmospheric cold plasma, low-pressure plasma could be used in the meat industry due to its advantageous properties such as the ability to treat large areas, even distribution in the vacuum chamber, uniformity of treatment and low process temperature.

### Scanning electron microscopy

*Escherichia coli* was grown in enriched broth for 24 h at 37 °C and then centrifuged at 5,000 *g* for 15 min. After centrifugation, the aqueous phase layer was collected and the pellet of bacterial culture obtained was removed to a cover glass and then exposed to helium and argon plasma for 2, 5 and 10 min according as described above. The control sample was not subjected to cold plasma treatment. Bacterial pellets were stored on cover glasses in 2.5 % glutaraldehyde overnight. SEM was used to investigate the cell structure of *E. coli* and examination was performed according to the protocol described by Kaliński et al. (2012).

### Statistical analysis

Each experiment was replicated three times and plate count analyses were made in quadruplicate for each sample (three independent experiments analyzed in quadruplicate). The effect of two independent categorical variables, such as time of exposure and type of gas being used, was evaluated. Data were analyzed by two-way factor analysis of variance (ANOVA) using Statistica 9 (StatSoft, Krakow, Poland). Differences between mean values were identified by Duncan’s test with a confidence level at *P* < 0.05.

## Results and discussion

### Antimicrobial activity

The effect of argon and helium plasma treatment on reduction of meat microbiota numbers is presented in Table 1. TNM, Y&M and P were initially around values of 4.96–5.23, 3.14–3.55, 3.65–4.13 CFU/cm^2^ respectively in both pork and beef muscles. The logarithmic reductions depend on time of exposure and type of gas being used. The highest inactivation efficiency was observed for helium plasma treatment. Y&M, TNM and P from pork muscle were reduced respectively about 1.90, 1.14, and 1.60 log cycle after 10 min exposure. Usage of argon plasma resulted in lower reduction of pork microbiota and was respectively 0.41, 0.77 and 1.20 log in the same time of treatment. It was also observed that TNM from beef were reduced of about 1.00 and 2.10 log after 5 and 10 min, respectively. These results indicate that increasing exposure time from 2 to 10 min significantly enhance decontamination effect of cold plasma treatment.

**Table 1:** Effect of cold plasma treatment on surface microbiota of meat. Values with different letters (a–e) within the same column differ significantly (*P* < 0.05).* TNM* Total number of microorganisms,* Y&M* yeasts and molds,* P* psychrotrophic microorganisms

Type of plasma	Exposure time (min)	Log reduction					
		TNM		Y&M		P	
		Pork	Beef	Pork	Beef	Pork	Beef
Helium	2	0.79 ± 0.10 c	0.55 ± 0.09 b	0.38 ± 0.11 b	0.25 ± 0.04 b	0.60 ± 0.10 b	0.19 ± 0.02 a
	5	0.94 ± 0.05 d	1.01 ± 0.03 c	0.60 ± 0.19 b	0.50 ± 0.06 c	0.81 ± 0.16 b	0.73 ± 0.21 b
	10	1.14 ± 0.03 e	2.09 ± 0.04 d	1.90 ± 0.02 c	0.98 ± 0.02 d	1.60 ± 0.02 d	1.48 ± 0.09 c
Argon	2	0 ± 0.01 a	0.16 ± 0.08 a	0 ± 0.01 a	0.04 ± 0.01 a	0.14 ± 0.02 a	0.23 ± 0.19 a
	5	0.34 ± 0.05 b	0.57 ± 0.06 b	0.40 ± 0.9 b	0.28 ± 0.09 b	0.50 ± 0.11 b	0.70 ± 0.30 b
	10	0.77 ± 0.08 c	0.56 ± 0.05 b	0.41 ± 0.8 b	0.50 ± 0.03 c	1.20 ± 0.01 c	1.32 ± 0.10 c

Different microorganisms showed variable sensitiveness to cold plasma treatments when inoculated onto the surface of agarized media (Tables 2, Table 3). Treatments resulted in reduction of tested bacteria counts except for *Listeria monocytogenes* ranging from 0.34 to 2.00 log orders of magnitude from an initial population of 10^3^ CFU/mL. The bacterium most susceptible to plasma exposure was *B. subtilis*. After 2 min of exposure a significant reduction of population of about 2.00 log was observed. After 5 min of exposure, helium plasma reduced *E. coli* and *Y. enterocolitica* by about 2.00 log. Similar results were obtained with argon plasma after 10 min. The *P. fluorescens* population was reduced by about 0.38 log using helium plasma for 5 min. A longer time of exposure caused a 1.97 log reduction of those bacteria. For argon plasma, a significant inactivation effect was also achieved within 10 min but the log reduction was lower (0.63 log).

**Table 2:** Effect of cold plasma treatment on Gram-positive bacteria inoculated onto the surface of agarized medium. Values with different letters (a–c) within the same column differ significantly (*P* < 0.05)

Type of plasma	Exposure time (min)	Log reduction				
		*Bacillus subtilis*	*Micrococcus luteus*	*Staphylococcus aureus*	*Listeria monocytogenes*	*Lactobacillus acidophilus*
Helium	2	1.92 ± 0.08 b	0.52 ± 0.21 b	0.64 ± 0.30 b	0 ± 0.01 a	0.20 ± 0.02 a
	5	2.00 ± 0.05 b	1.06 ± 0.11 c	1.98 ± 0.04 c	0.06 ± 0.02 a	0.53 ± 0.11 b
	10	1.97 ± 0.05 b	1.48 ± 0.09 c	2.02 ± 0.07 c	0.11 ± 0.07 a	0.69 ± 0.08 b
Argon	2	1.79 ± 0.18 b	0.01 ± 0.01 a	0.08 ± 0.05 a	0 ± 0.00 a	0 ± 0.00 a
	5	1.98 ± 0.05 b	0.34 ± 0.09 b	0.88 ± 0.12 bc	0 ± 0.01 a	0 ± 0.01 a
	10	2.00 ± 0.07 b	0.89 ± 0.12 c	0.96 ± 0.16 bc	0.09 ± 0.05 a	0.17 ± 0.1 a

**Table 3:** Effects of cold plasma treatment on Gram-negative bacteria inoculated onto the surface of agarized medium. Values with different letters (a–c) within the same column differ significantly (*P* < 0.05)

Type of plasma	Exposure time (min)	Log reduction		
		*Escherichia coli*	*Yersinia enterocolitica*	*Pseudomonas fluorescens*
Helium	2	0.44 ± 0.13 b	1.98 ± 0.02 c	0.01 ± 0.01 a
	5	1.99 ± 0.04 c	1.97 ± 0.04 c	0.38 ± 0.13 b
	10	2.01 ± 0.02 c	2.00 ± 0.01 c	1.97 ± 0.06 c
Argon	2	0.01 ± 0.01 a	0.34 ± 0.09 b	0.01 ± 0.00 a
	5	0.49 ± 0.11 b	1.12 ± 0.07 c	0.01 ± 0.00 a
	10	1.99 ± 0.03 c	1.96 ± 0.04 c	0.63 ± 0.14 b

No significant differences in *Listeria monocytogenes* counts were noted between the control sample and samples treated with helium and argon plasma. *Lactobacillus acidophilus* showed similar resistance against argon plasma but use of helium enabled a significant reduction of more than 0.50 log after 5 min. The results clearly indicate that helium plasma treatment is more effective than argon plasma in inactivation of microorganisms if comparing the same time of exposure. Although an antimicrobial activity of cold plasma was confirmed, in some cases the use of other methods of decontamination to enhance microbiological safety of meat seems justified. Combination of food preservation methods is known as hurdle technology and was described by Leistner and Gould (2002). Previous study has proved the antibacterial activities of chitosan films, therefore application of low-pressure plasma combined with edible coatings and/or films may be more efficient in food preservation as well as reducing the physical changes in fresh products (Ulbin-Figlewicz et al. 2014).

According to Moisan et al. (2002), UV radiation and erosion processes provide a mechanism of cold plasma sterilization. Inactivation of the DNA and RNA or etching of cell wall/membrane is affected by UV radiation, especially in low-pressure plasma because the content of UV photons can be considerable. Erosion can be attributed to the etching activity of radicals and charged particles (Moisan et al. 2002). Low-pressure plasma decontamination processes use non-toxic gas mixtures (for instance Ar, H_2_, O_2_, N_2_ etc.) (Rossi et al. 2008). Depending on the process gas, various chemically reactive species are generated and these show different effects on microorganisms (Fricke 2012). The sterilization effectiveness of helium and argon plasma may be attributed to the fact that the ionization potential of helium (24.6 eV) is higher than that of argon (15.8 eV). In contrast to helium, argon plasma does not generate substantial quantities of elements possessing high ionization energies (Okino et al. 1996). In addition, noble gas plasmas produce very intense UV radiation, especially in the helium plasma (Weidner et al. 1998). Radicals could cause etching of the cell wall/membrane, oxidation of proteins, DNA, RNA and enzymes, while charged particles cause etching, perforation of the cell wall and electroporation. Pulses of electric field could also lead to irreversible damage to membranes (Fricke 2012). The lifetime of the mentioned reactive species at reduced pressure is much longer in comparison to atmospheric plasma (Shintani et al. 2010).

A high resistance of *B. subtilis* to a variety of treatments, including heat, UV radiation, and oxidizing agents such as hydrogen peroxide, has been reported by many authors (Setlow 2006; Hanlin et al. 1985; Popham et al. 1995). Despite the fact that bacterial spores are more resistant than vegetative cells to physical and chemical treatments, destroying spores by exposure to low-pressure plasma is possible as a result of UV photons passing through the spore-protecting coats and damaging the DNA within (Moisan et al. 2002; Rossi et al. 2008). However, erosion of the spore surface as an additional mechanism must be provided to make it easier for the photons to reach the DNA (Moisan et al. 2002). von Kuedell et al. (2010) observed that spores of *Bacillus atrophaeus* were reduced by at least four orders of magnitude under optimized low-pressure argon plasma. Lerouge et al. (2000) indicated that spore mortality depends on plasma gas compositions (O_2_, O_2_/Ar, O_2_/H_2_, CO_2_, and O_2_/CF_4_). The highest efficiency was seen with O_2_/CF_4_ plasma, giving a 5 log decrease within 7.5 min. These latter authors also indicated that etching contributes to spore mortality. Similar results were obtained by Roth et al. (2009), who indicated that *B. subtilis* spores are inactivated efficiently by a combination of protein inactivation and DNA damage due to the dominant role of UV radiation and erosion processes.

Gram-positive bacteria are more resistant than Gram-negative bacteria due to differences in cell-wall structure. Thus, longer treatment times may be necessary to damage the outer cell membrane of Gram-positive bacteria and cause cell lysis (Weltmann et al. 2012). Purevdorj et al. (2002) found that growth of *E. coli* was reduced by about 4.47, 5.19 and 6.29 log cycle after 30 min of argon plasma treatment with increasing microwave power density in the range of 1.47, 2.63 and 4.21w/cm^3^, respectively. A high sensitivity of *E. coli* to N_2_O plasma treatment was observed by Chau et al. (1996); the time required for killing those bacteria is 2 min. Chau et al. (1996) also found that *P. fluorescens* exhibits the greatest resistance to treatment, with the time of inactivation being 10 min (Chau et al. 1996). In the last few years, atmospheric pressure plasmas have been the object of many investigations. Kim et al. (2011) observed reduction of *Listeria monocytogenes*, *E. coli* and *Salmonella typhimurium* on sliced bacon treated with helium and helium/oxygen plasmas of about 1–2 log cycles and 2–3 log cycles, respectively. Inactivation of *E. coli* on almonds, apples, pork has also between described by other authors (Deng et al. 2007; Niemira and Sites 2008; Moon et al. 2009). Despite these promising results, the implementation of atmospheric plasma in the meat industry is limited due to the small area that can be targeted for treatment. Depending on the experimental parameters of cold plasma treatment, the antibacterial effect is different. Rossi et al. (2008) investigated the effect of low-pressure plasma on depyrogenation of bacterial endotoxins such as lipopolysaccharides (LPS), which constitute a major part of the outer cell wall of Gram-negative bacteria. After 5 min exposure, a significant reduction in LPS bioactivity of about 2 orders of magnitude was observed. These authors also indicated that the efficiency of depyrogenation of LPS and Lipid A was accelerated significantly by the enhancement of hydrogen content in the plasma gas mixture. Selcuk et al. (2008) observed a significant reduction of 3 log for both *Aspergillus* spp. and *Penicillium* spp. artificially contaminated on seed surfaces within 15 min of SF_6_ plasma treatment time. Inactivation of these pathogenic fungi depends on seed surface, plasma gas type, plasma treatment time, and microbial population density. Seed quality was not affected by air and SF_6_ plasma treatment. The percentage germination rate of wheat or bean seeds treated with low-pressure plasma did not differ significantly compared to an untreated sample. According to Pignata et al. (2014), both oxygen and argon low-pressure plasma caused a reduction of *Aspergillus brasiliensis* of about 3.5 log cycles after 30 min of exposure, while a mixture of both gases resulted in a 5.4 log reduction. For the reduction of *E. coli*, a shorter time of treatment was required because of its higher sensitivity to cold plasma. Treatment for 30 s and 60 s resulted in a 4 log reduction with argon and oxygen plasma, respectively.

Lerouge et al. (2001) indicated gas composition as a determining factor in the effectiveness of plasma. Gas flow rate and pressure, the type of generator design and power, all play an important role as well. Higher electron and ions density caused by rising power, in synergy with ultraviolet photons, led to better destruction of bacterial cells (Bol’shakov et al. 2004). On the other hand, issues related to treated material need to be taken into consideration. Packaging, the nature and quantity of substrates, and the temperature and nature of microorganisms are highly relevant to the decontamination effect (Lerouge et al. 2001).

One of the major sources of food contamination and clinical infection are pathogens able to form biofilms. This is a complex process in which genetic mechanisms and the properties of both substratum and bacterial cell surfaces, as well as environmental factors, are involved (Shi and Zhu 2009). The relationship between stress and biofilm formation was confirmed by Zhang et al. (2007). They indicated that *E. coli* produce more biofilm in response to adverse environmental conditions such as acidic pH, oxidative stress (H_2_O_2_), heavy metals [Cd(II)], and cold shock (22 °C). Surface structures of bacteria such as flagella, pili and curli are used to initiate biofilm formation (Pratt and Kolter 1998; Zhang et al. 2007). Because of the different properties of biofilms, resistance to conventional sterilization methods is higher. In addition, usage of chemical compounds (e.g., ethylene oxide or chlorine), high temperature and radiation have some limitations relating to thermolabile products or human health. Scientists are looking for new methods to biofilm inactivation, and cold plasma technology could be one of them (Brelles-Mariño 2012). Therefore investigation into the effect of low-pressure plasma on biofilm inactivation needs to be considered in future.

### Scanning electron microscopy

**Fig 2a–e:**
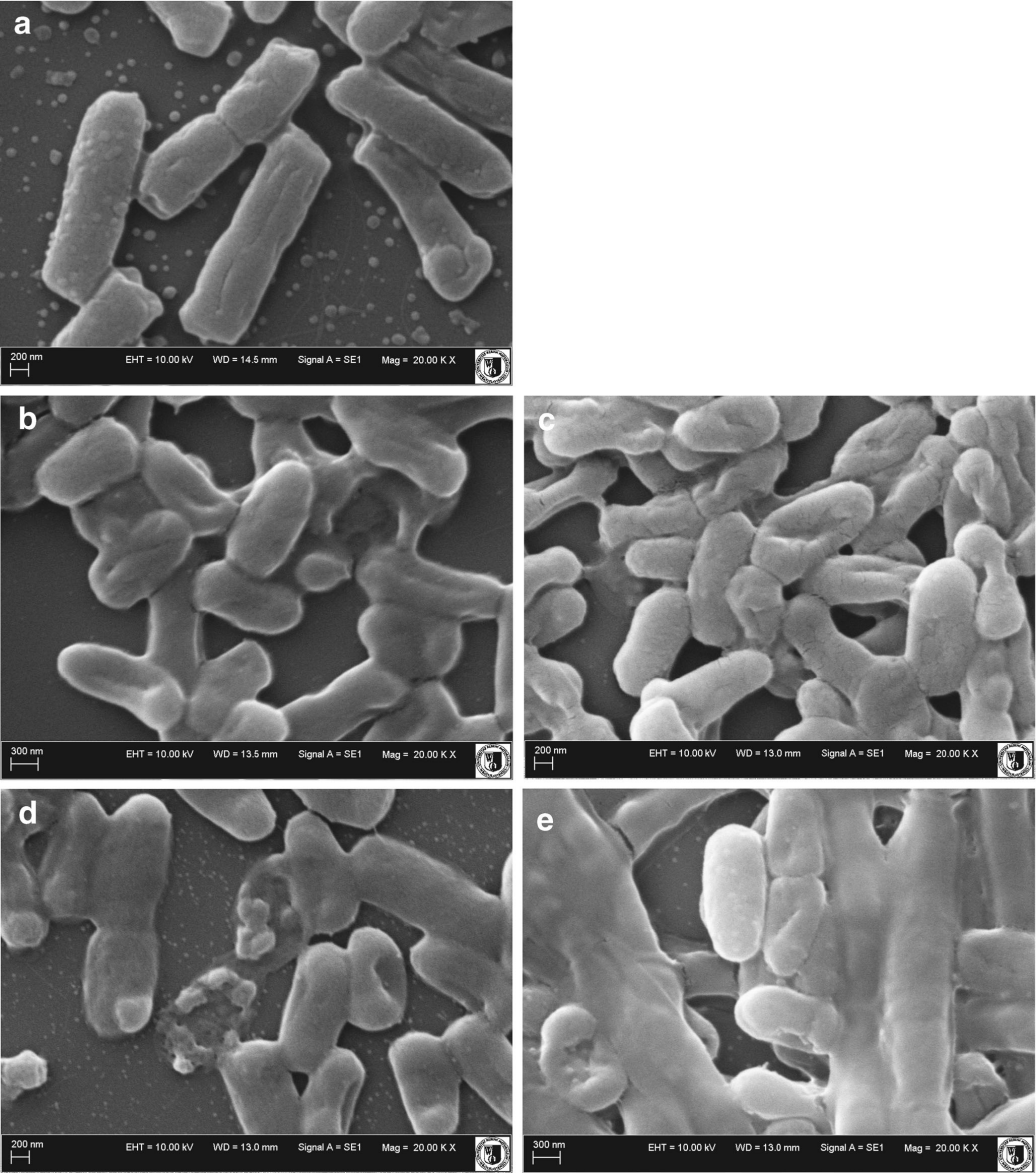
Scanning electron microscopy (SEM) images of *Escherichia coli*. **a** Untreated control. **b** After helium plasma exposure for 2 min. **c** After argon plasma exposure for 2 min. **d** After helium plasma exposure for 10 min.** e** After argon plasma exposure for 10 min

Scanning electron microscopy (SEM) was used to examine morphological changes of *E. coli* bacteria exposed to cold plasma treatments (Fig. 2). Argon and helium plasmas treatment for 2 min caused shrinkage in a bacterial cell wall, suggesting a bacteriostatic effect. A bactericidal effect was evidenced, since lysis of cells subjected to helium plasma was observed (Fig. 2d). Some of the cells were completely destroyed and, as a result, a bactericidal effect was noted. Disruption of *E. coli* cells exposed to argon plasma for 10 min was also observed. These changes in the morphological structure of the cell indicate that argon plasma treatment induces lysis, but we are still an early stage in this field. SEM results are in agreement with microbiological analysis. Reduction in the log counts of the microbes subjected to treatment increases with increased exposure time, because damage to bacterial cells is greater.

Hong et al. (2009) observed severe cytoplasmic deformations and leakage of the bacterial chromosome in cells treated with atmospheric plasma. Disruptions and cell lysis of *E. coli* exposed to Ar-NO plasma treatment was also demonstrated by Hueso et al. (2008).

**Fig. 3:**
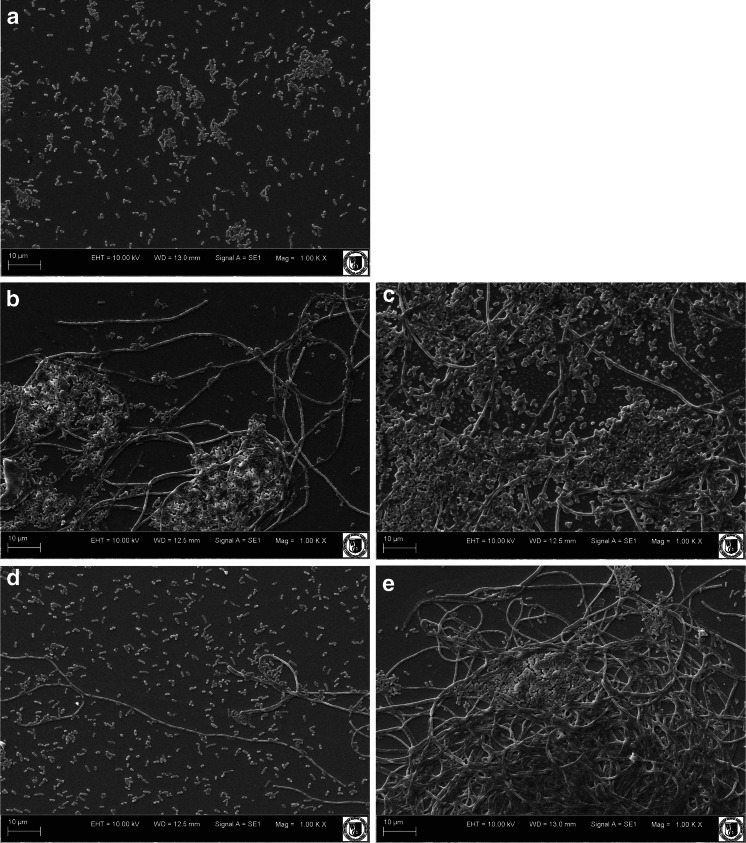
SEM images of aggregated cells of *Escherichia coli*. **a** Untreated control. **b** After helium plasma exposure for 2 min. **c** After argon plasma exposure for 2 min. **d** After helium plasma exposure for 10 min.** e** After argon plasma exposure for 10 min

Untreated *E. coli* cells appeared intact and separated from each other (Fig. 3a), while bacterial cells treated with low-pressure plasma appeared to be aggregated (Fig. 3b,e). It can be hypothesized that the observed differences are caused by modification of the surface properties of cells exposed to plasma treatment. The aggregation of bacterial cells as a response to stress due to antibacterial agents was also reported by other authors. Tyagi and Malik (2012) observed morphological alterations in *E. coli* cells treated with lemon grass oil. They reported that cytoplasmic material of the bacterial cells had leaked and the aggregate cells appeared as sludge. According to Tang et al. (2007), both *E. coli* and S*hewanella oneidensis* aggregated after addition of C_60_-NH_2_ (fullerene compound). They indicated that aggregation was associated with oxidative stress and could represent protection mechanism.

## Conclusions

The results of this study showed the effectiveness of low-pressure plasma treatment for decontamination of meat. Use of helium plasma results in a higher reduction of microbes compared to argon plasma treatment. Loss of viability of bacteria is explained by serious damage to cell morphology. The effectiveness of cold plasma varies with experimental parameters, the subject material and the nature of the microorganism. The results suggest that application of low-pressure plasma is a promising treatment for inactivation of surface microbiota of meat.

## Footnotes

1 ISO 18593:2004. Microbiology of food and animal feeding stuffs. Horizontal methods for sampling techniques from surfaces using contact plates and swabs

2 ISO 17604:2003 Microbiology of food and animal feeding stuffs. Carcass sampling for microbiological analysis

3 ISO 2293:1988 Meat and meat products. Enumeration of microorganisms. Colony count technique at 30° C

4 ISO 17410:2001 Microbiology of food and animal feeding stuffs. Horizontal method for the enumeration of psychrotrophic microorganisms

5 ISO 21527–1:2008 Microbiology of food and animal feeding stuffs. Horizontal method for the enumeration of yeasts and moulds. Part1: colony count technique in products with water activity greater than 095
